# Exposure to Temozolmide in the First Trimester of Pregnancy in a Young Woman With Glioblastoma Multiforme

**DOI:** 10.4021/wjon570w

**Published:** 2013-01-04

**Authors:** Bernadette Nolan, Madhuri Balakrishna, Mathew George

**Affiliations:** a University of Newcastle/University of New England Joint Medical Program, Australia; b North West Cancer Centre, Hunter New England Area Health Service, Australia

**Keywords:** Temozolomide, Glioblastoma multiforme, Early pregnancy, First trimester pregnancy, Teratogenicity, Malformations, Complications

## Abstract

Literature on the outcome of pregnancy with first trimester exposure to Temozolomide is limited. We describe the case of a young woman with Glioblastoma Multiforme who was exposed to Temozolomide during her first trimester of pregnancy and subsequently delivered a healthy term newborn. At six months of age, the child remains healthy with no evidence of Temozolomide related effects.

## Introduction

Glioblastoma multiforme (GBM), a grade IV diffuse astrocytoma, is a rapidly progressive brain tumour with a median survival of less than two years [1]. Clinical manifestations of GBM are similar to other brain tumours and depend on extent and location. The current incidence is estimated at 3.96 cases/100,000 person-years, with a 1.6:1 male:female ratio [2].

The management approach to GBM involves initial surgical resection, followed by adjuvant combined chemotherapy and radiotherapy, and subsequent chemotherapy [3]. Temozolomide is the current first-line chemotherapy agent of choice, and when combined with radiotherapy, has been shown to prolong overall survival with statistically significant results [4].

Temozolomide is an oral alkylating chemotherapy agent, currently classed as a category D drug in pregnancy [3]. Adverse effects include mild myelosuppression, gastrointestinal upset, fatigue and anorexia [5]. Complications of exposure during pregnancy observed in animal studies include teratogenicity and foetal toxicity [6]. However, there have been no adequate and well-controlled human studies [3]. Current recommendations for both men and women include stopping Temozolomide for six months prior to conception [6].

We report here a case of first trimester exposure to Temozolomide that is of interest as it resulted in an uncomplicated pregnancy and normal infant despite the category D status in pregnancy of this drug.

## Case Report

A previously well 23-year-old female with no significant past medical history presented to her local general practitioner in a rural town in NSW, Australia, with a six week history of severe left sided headache. Additional symptoms included nausea and vomiting, and more recently, blurred vision, confusion and memory lapse. Neurological examination did not reveal any abnormalities. CT scan of the brain performed at the nearby rural referral hospital showed a large heterogenous mass lesion, approximately 6.4 cm in diameter, involving the left frontal lobe and left basal ganglia. She was transferred to a regional centre where MRI of the brain revealed a large semi-necrotic lesion within the left frontal lobe and some satellite cells.

She underwent uncomplicated stereotactic-guided craniotomy and resection of the left frontal lobe tumour. Histopathology confirmed a diagnosis of glioblastoma multiforme. She was subsequently treated with concurrent radiotherapy and Temozolomide for six weeks, completing this therapy in November 2010. She was then treated for six months with adjuvant Temozolomide and completed this course in mid-May 2011. At the commencement of treatment, the patient was advised to avoid pregnancy due to potential risks to the foetus.

Following the completion of her chemotherapy, the patient was found to be pregnant, with conception calculated to be early May. Thus, the foetus was exposed to the final cycle of Temozolomide at approximately two weeks of gestation. The patient was managed by the High Risk Pregnancy team at the regional centre throughout her pregnancy and subsequently delivered a healthy 3,220 gram infant at term in January 2012. There were no neonatal complications and currently at six months of age, the infant appears to be unharmed by the exposure to Temozolomide during the first trimester.

## Discussion

Cancer is the second most common cause of mortality in women of reproductive age [7].

Due to the poor understanding of pharmacokinetics and issues surrounding safety of chemotherapy agents in pregnancy, trials examining the use of chemotherapy agents in pregnancy are limited. However, a literature review of the use of antineoplastic agents conducted by Selig et al [7] has demonstrated that antimetabolites and alkylating agents have the highest rates of adverse pregnancy outcomes (APOs), more common with exposure in the first trimester. APOs include congenital malformations, functional defects, blood or electrolyte abnormalities, stillbirths, miscarriages and death [7]. In addition, they also found that APOs were more common when single agent chemotherapy was used [7].

As previously mentioned, there have been no studies conducted surrounding the use of Temozolomide in pregnant women [6]. However, the outcome of pregnancy with first trimester exposure to Temozolomide has been previously described as part of a case series of three patients by Blumenthal et al (2008) [5]. All three women with pre-existing malignant glioma and receiving anticonvulsant medication were inadvertently exposed to Temozolomide during their first trimester of pregnancy. They all had uneventful pregnancies and delivered healthy full-term newborns without evidence of congenital malformations. Similar to the case we describe here, Temozolomide was discontinued. However, anticonvulsant therapy was continued throughout the pregnancies in the case series.

With this case report, we hope to add to the literature an additional example of an uncomplicated pregnancy and normal foetal outcome with first trimester exposure to Temozolomide. This case report is unique from existing literature in that it describes Temozolimide as a single agent, with no confounding effects of other potentially teratogenic agents. However, we are limited by the short follow-up and inability to assess potential late effects of Temozolomide exposure to the child.

### Summary

Literature on the outcome of pregnancy with first trimester exposure to Temozolomide is limited. We describe the case of a young woman with Gioblastoma Multiforme who was exposed to Temozolomide during her first trimester of pregnancy and subsequently delivered a healthy term newborn. At six months of age, the child remains healthy with no evidence of Temozolomide related effects.

### Key points

1) Temozolomide is listed as a category D drug in pregnancy. 2) There is limited literature on first trimester exposure to Temozolomide and the outcomes of these pregnancies. 3) The effects of Temozolomide during pregnancy have never been formally studied.
